# Supplementary material for: The interactome of intact mitochondria by cross-linking mass spectrometry provides evidence for coexisting respiratory supercomplexes
Source: Mol Cell Proteomics. 2017 Dec 8;17(2):216–32. doi: 10.1074/mcp.RA117.000470 (PMC5795388; doi:10.1074/mcp.RA117.000470)
Supplement: Supplemental Data [file supp_RA117.000470_133922_0_supp_23978_fzffwf.zip › spectra_annotation/mito_spectra_annotation/470-1-20-1-7-1.pdf]

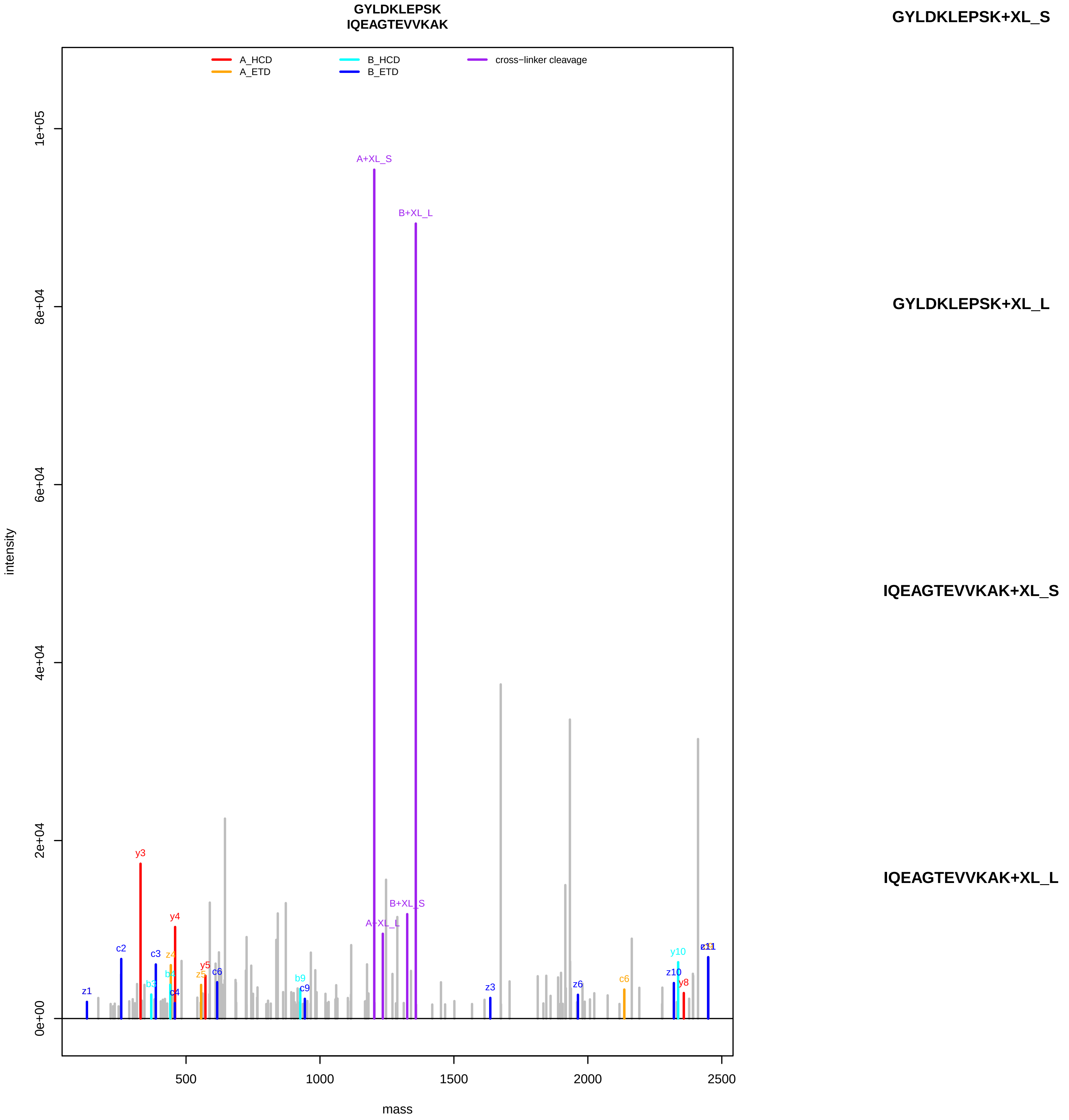
GYLDKLEPSK
IQEAGTEVVKAK
A_HCD
A_ETD
B_HCD
B_ETD
cross−linker cleavage
intensity
mass
0e+00
2e+04
4e+04
6e+04
8e+04
1e+05
500
1000
1500
2000
2500
A+XL_S
B+XL_L
B+XL_S
A+XL_L
y3
y4
y5
y8
y10
z1
c2
c3
z4
b3
b4
c4
z5
c6
b9
c9
z3
z6
c6
z10
z11
GYLDKLEPSK+XL_S
GYLDKLEPSK+XL_L
IQEAGTEVVKAK+XL_S
IQEAGTEVVKAK+XL_L
